# The Molecular Link between Obesity and the Endometrial Environment: A Starting Point for Female Infertility

**DOI:** 10.3390/ijms25136855

**Published:** 2024-06-22

**Authors:** Francesca Gonnella, Fani Konstantinidou, Marisa Donato, Daniela Maria Pia Gatta, Alessia Peserico, Barbara Barboni, Liborio Stuppia, Warren B. Nothnick, Valentina Gatta

**Affiliations:** 1 Department of Psychological Health and Territorial Sciences, School of Medicine and Health Sciences, “G. d’Annunzio” University of Chieti-Pescara, 66100 Chieti, Italy; fgonnella@unite.it (F.G.); fani.konstantinidou@unich.it (F.K.); mdonato1@unite.it (M.D.); liborio.stuppia@unich.it (L.S.); 2 Unit of Molecular Genetics, Center for Advanced Studies and Technology (CAST), “G. d’Annunzio” University of Chieti-Pescara, 66100 Chieti, Italy; 3 Department of Bioscience and Technology for Food, Agriculture and Environment, University of Teramo, 64100 Teramo, Italy; apeserico@unite.it (A.P.); bbarboni@unite.it (B.B.); 4 Department of Pathology, SS Annunziata Hospital, 66100 Chieti, Italy; daniela.gatta@unich.it; 5 Department of Cell Biology and Physiology, University of Kansas Medical Center, Kansas City, KS 66160, USA; wnothnic@kumc.edu; 6 Department of Obstetrics and Gynecology, University of Kansas Medical Center, Kansas City, KS 66160, USA; 7 Department of Cancer Biology, University of Kansas Medical Center, Kansas City, KS 66160, USA; 8 Center for Reproductive Sciences, University of Kansas Medical Center, Kansas City, KS 66160, USA

**Keywords:** obesity, endometrium, gene expression, endometrial receptivity, endometriosis

## Abstract

Female infertility constitutes a growing health problem in developing countries and could be associated with several possible causes including reproductive disorders, congenital malformations, infections and hormonal dysfunction. Nonetheless, a series of additional factors can also negatively impact female fertility and are represented by chronic exposure to environmental pollutants, stress, unhealthy lifestyle choices such as cigarette smoking and, among others, obesity. Excess weight is associated with several chronic diseases, and growing evidence demonstrates that it can compromise reproductive physiology due to its influence on endometrial gene expression and receptivity. Thus, the current review of the literature mainly focused on how obesity can impair uterine receptivity, mostly from a molecular point of view throughout the window of implantation (WOI) period at an endometrial level. It was also highlighted that an obesity-related increase in adipose tissue may lead to a modulation in the expression of multiple pathways, which could cause a hostile endometrial environment with a consequent negative impact on the uterine receptivity and the establishment of pregnancy. Thanks to the use of the endometrial receptivity assay (ERA), a specific microarray that studies the expression of a series of genes, it is now possible to evaluate the endometrial status of patients with infertility problems in a more detailed manner. Moreover, female fertility and endometrial receptivity could be affected by endometriosis, a chronic benign gynecological disease, whose cause-and-effect relationship to obesity is still uncertain. Therefore, further investigations would be required to better elucidate these mechanisms that govern embryo implantation and could be potentially useful for the generation of new strategies to overcome implantation failure and improve the pregnancy rates in obese women.

## 1. Introduction

Worldwide, an average of 10 to 15% of couples of reproductive age are affected by infertility, a multifactorial pathological state. Female infertility constitutes a growing health problem in developing countries, given the tendency to postpone pregnancy beyond 35 years of age, which may considerably reduce fertility rates. There are several possible causes associated with female infertility, including infections, reproductive disorders, congenital malformations, hormonal dysfunctions and tubal or uterine malformations. Non-modifiable risk factors, such as aging and genetic mechanisms, are also increasingly linked to the onset of infertility. Moreover, female fertility can be negatively influenced by factors associated with lifestyle, such as stress, chronic exposure to environmental pollutants, competitive sports, cigarette smoking, high consumption of caffeine or alcohol, unhealthy eating habits or obesity [1,2]. Obesity has a complex etiology, but a high percentage of individuals are affected by diet-induced obesity (DIO) or, more specifically, by the overconsumption of a diet high in fat. In humans, a high-fat diet (HFD) takes up 30% to 70% of the total caloric intake and leads to increased lipid deposition in both adipose and non-adipose tissues. A high dietary fat intake has been found to reduce female fertility and the functioning of the hypothalamic–pituitary–ovarian (HPO) axis, whether or not obesity develops [3]. Therefore, the increased risk of sub-fertility and infertility in obese women may be subsequently related to aberrations in folliculogenesis, oocyte maturation, ovulation and even impaired endometrial development and receptivity [4,5]. The endometrium is of particular interest considering its role, as one of the most dynamic tissues in the human body, in embryo implantation and the imminent development of pregnancy [6]. The success of embryo implantation relies on the synchronization of a viable embryo with a receptive endometrium [7]. For this reason, the window of implantation (WOI) has been thoroughly examined. WOI is a critical time period during which the human endometrium is most receptive to the embryo. Assisted reproductive technology (ART) outcomes can also be greatly improved by individualized embryo transfers and accurate WOI determination. The negative effect of weight excess on endometrial receptivity during the window of implantation (WOI) has been well established, indicating that nutritional habits could significantly affect the endometrial environment [8]. In fact, numerous studies have reported that elevated BMIs may impair endometrial receptivity by inducing significant endometrial transcriptomic differences between obese and non-obese women, inevitably contributing to lower implantation and increased miscarriage rates [9,10]. In this context, currently, many transcriptomic analyses of genes linked to embryonic implantation and endometrial proliferation are performed via the endometrial receptivity assay (ERA), or ER Map, a useful commercially available molecular tool able to evaluate the receptivity of the human endometrium [11]. Overall, the existing literature sustains that obese women may exhibit differential gene expression in the endometrium in association with endometrial cancer [12] and angiogenesis [13], as well as an inflammatory response and decidualization [14], highlighting the importance of this kind of investigation to better comprehend the receptivity and, potentially, embryo implantation. To date, not all contributing factors able to interfere with endometrial receptivity are well known, but, apart from inflammatory events, they are often associated with endocrine causes, thin endometria, polyps, septa, fibroids, immunologically mediated disturbances, pre-eclampsia [15], cardiovascular risk factors such as dyslipidemia and hypertension, and diabetes [16] contributing to a series of reproductive problems [17]. The diagnosis of one of the most frequent gynecological conditions, such as endometriosis, has also been evaluated in subjects with higher BMIs. Endometriosis is a chronic, benign and estrogen-dependent disease characterized by the presence of endometrial tissue in the peritoneal cavity. This inflammation affects about 2–10% of women of reproductive age and 2–5% of postmenopausal women on hormone therapy, but the number of unreported cases is estimated to be higher [18,19,20,21]. However, the cause–effect relationship between obesity and endometriosis and its role in endometrial receptivity remains uncertain. 

Thus, the purpose of this review is to highlight how obesity can have a negative impact on female reproductive health, by specifically focusing on its effect on the endometrial gene expression pathways, such as inflammation, oxidative stress and the immune response during the window of implantation and the subsequent compromised endometrial receptivity. Endometrial gene expression is influenced both directly by obesity and indirectly through the effect of obesity on the oviduct and ovarian hormones. In addition, it also aims to shed light on its possible connection to one of the most diffused gynecological conditions such as endometriosis, potentially underlining a cause–effect relationship between them.

## 2. The Effect of Obesity on the Endometrium

### 2.1. Impact of Obesity on Endometrial Gene Expression and Receptivity during the Preimplantation Period

It has been widely documented that obesity is able to have potential effects on gene expression in a variety of reproductive cells and tissues, influencing processes such as gene expression, endometrial development and receptivity, ovulation and embryo implantation. It has also been demonstrated that the endometrial physiology and gene expression undergo significant changes in obese subjects as a consequence of transcriptional changes [1,5] (Table 1).

For instance, a study has been performed during the implantation window on endometrial samples obtained from overweight and obese women, stratified and analyzed according to their IVF results in a pregnancy (PG; *n* = 5) or non-pregnancy group (non-PG; *n* = 9). An increase in the expression of genes involved in the pathways related to the immune response, ROS production and inflammation was detected in the patients who had not yet conceived. More specifically, higher levels of the *SDF1* gene, also known as *CXCL12*, and its receptor *CXCR4*, as well as of two classes of T-helper lymphocytes, proinflammatory *TH1* and anti-inflammatory *TH2*, were evidenced in non-pregnant endometrial samples, potentially suggesting a compromised embryo implantation. In addition, the pathway of the *NRF2*-mediated oxidative stress response, that exerts a key role in the protection of cell function by regulating antioxidant enzymes, and the pathway of the nuclear factor of activated T cells (*NFAT*) family, which is involved in the regulation of immune response, were found to be upregulated in women with a non-receptive endometrium, presumably due to the impaired inflammatory state of the latter. Considering all the above, the over-activity of these pathways in women who did not conceive, could cause a hostile environment in the endometrium that exerts a negative impact on the establishment of pregnancy [22].

The effect of obesity on gene expression during the implantation period has also been highlighted in ovine models. More specifically, differentially expressed genes (DEGs) (*n* = 699) were detected in the endometrium of obese compared to lean sheep during the peri-implantation period, with 171 of them resulting in being downregulated and 498 upregulated [23]. Different cellular and reproductive processes were linked to these genes, including cellular metabolic processes, immune system processes, placental development and function, cell death, growth, and adhesion to the endometrium. Among the DEGs identified in the comparison between obese and lean ovine endometria, the upregulation of osteoponin, also known as the *SPP1* gene, a mediator of trophoblast–endometrial adhesion and angiogenesis during the peri-implantation period of early gestation, suggests an advanced progression to the implantation stage in obese animals. In addition, other upregulated genes identified in obese ewes are the placental growth factor (*PGF*) and nitric oxide synthase 2 (*NOS2*), transcripts encoding proteins with a fundamental role in the regulation of vasculogenesis and angiogenesis in the peri- and post-implantation period. These findings could indicate that the increased expression of proangiogenic factors in the endometrium of obese ewes leads to an enhanced blood flow and thus an increment of nutrient supply to the offspring, potentially explaining the abnormal growth of lambs born to obese mothers [24,25,26]. Thus, it could be suggested that alterations in uterine transcript profiles during early embryogenesis may be a mechanism responsible for developmental reprogramming due to the maternal obesity exposure in utero which leads to an alteration of conceptus implantation and placentation. Moreover, these observations provide evidence that the endometrial gene expression is modified during the peri-implantation period in ways that could potentially compromise the gestation later and/or affect the offspring’s health after birth [23]. 

Another study carried out on equine endometrial progenitor cells (Eca EPCs) obtained from obese mares reported an increased percentage of early apoptosis and loss of mitochondrial dynamics as well as a senescence-associated phenotype. These cells showed an increased expression of the pro-apoptotic markers (BAX, CASP9, P53, P21), osteopontia (OPN), phosphatidylinositol 3-kinase (PI3K) and protein kinase B (AKT), as well as a decreased expression of B-cell lymphoma 2 (BCL-2), forkhead box P3 (FOXP3), sirtuin 1 (SIRT1) and mitofusin 1 (MFN1). These genes are critical for maintaining cellular homeostasis. The balance of apoptosis and proliferation is critical for endometrial regeneration during the reproductive cycle. Moreover, the imbalance of these pathways in obese mares may contribute to adenomyosis. These changes suggest that the endometrium of obese subjects may be less prone to cyclic changes or to establishing an optimal environment for the embryo [27]. Additionally, Shankar et al. [28] conducted a study on the uterus of obese compared to lean rats (*n = 10 per group*) in order to highlight how maternal obesity alters the uterine gene expression during the WOI and how it influences their respective peri-implantation blastocyst (*n = 30 per group*) with the purpose to identify the earliest changes associated with maternal obesity. The uterine gene expression profiles of obese and lean dams showed a distinct signature for the genes regulating inflammation and lipid metabolism. In particular, the analysis revealed that the 403 transcripts involved in the immune response, inflammation and cytokine/chemokine signaling of the uterus’s expression profile were differentially expressed in obese rats. Among the inflammatory-related genes studied, an upregulation of chemokines like *Ccl2*, *Ccl5*, *Ccl7* and *Cxcl10* and their related regulators, *Tlr2*, *Cd14* and *Ccr1*, was evidenced, confirming that the major modifications in utero concerned the immune response. Furthermore, data showed the over-expression of genes associated with lipid metabolism such as lipoprotein lipase (*Lpl*), fatty-acid-binding protein-4 (*Fabp4*) and *Cd36*, as well as insulin resistance, such as the retinol adipokine-binding protein-4 (*Rbp4*), in the uterus of obese rats. Consequently, it was demonstrated that the exposure to maternal obesity could lead to uterine lipid deposition and extensive proinflammatory gene expression changes during the WOI, suggesting that both the blastocyst and uterus of obese dams could be negatively affected by the intrauterine environment. 

The maternal body composition can also have a significant impact on the development of obesity and metabolic syndrome in adult offspring. An analysis carried out on the endometria of normal-weight (n = 9) and obese mares (n = 6) and on their preimplantation embryos (n = 5 per group) was aimed to determine the effect of maternal obesity in the absence of a high-fat diet on the endometrium and blastocysts [29]. It was observed that the endometrium of obese mares displayed a marked increase in the mRNA levels of inflammatory cytokines, including tumor necrosis factor-α (*TNFA*), a cytokine involved in inflammation, interleukin-1β (*IL1B*), which constitutes an essential mediator of maternal-placental immunotolerance during implantation and transcription factor-6 (*ATF6*), a main factor responsible for the release of *TNFA*, *IL1B* and *IL6*. Moreover, embryos from obese mares exhibit an upregulation of genes involved in inflammation, lipid homeostasis as well as oxidative, mitochondrial and endoplasmic reticulum (ER) stress. The results obtained could indicate that the increased adiposity in mares alters the uterine environment and is associated with the increased inflammation in the mares’ endometrium. Furthermore, maternal obesity could be associated with the altered expression of genes related to lipid homeostasis and mitochondrial, oxidative and ER stress in the embryos of obese mares. These alterations may affect prenatal programming, but further investigation is needed to determine how they impact on the developing offspring. 

Another study conducted on obese women reported elevated levels of the Advanced Glycation End Products (AGEs) in the uterine cavity and an increase in the AGE receptor (RAGE) in endometrial glandular epithelial cells, furnishing a mechanism for AGE-RAGE-mediated inflammatory signaling [15]. This could be of interest considering that previous studies had suggested that AGE-RAGE regulation relates to apoptosis, inflammatory reaction, oxidative stress, and angiogenesis through vascular endothelial growth factor activation [30]. An in vitro experiment was subsequently carried out adding to the primary stromal cells’ levels of AGEs equimolar with those within the uterine cavity (obese AGEs). As a result, these obese AGEs altered the endometrial epithelial cell function, detrimentally impacted the decidualization and promoted the nuclear accumulation of NFκB. These data indicate that the primary function of stromal cells is deeply influenced by obese AGEs, reducing their decidualization ability in the endometrium of obese women and thus contributing to the decrease in fertility. This study, therefore, supports a role of elevated AGEs in the pathophysiology of pre-eclampsia, explaining why obese women have a higher risk of pre-eclamptic pregnancies [15].

Based on these premises, it could be argued that obesity may contribute to the development of inflammation through the significantly higher expression of several mediators, possibly compromising embryo implantation.

Another crucial aspect for successful implantation and early pregnancy is decidualization, a hormonally mediated process by which endometrial stromal cells (ESCs) proliferate and differentiate into decidual cells. Therefore, the research carried out on both mouse and human endometria was designed to investigate the effect of diet-induced obesity on ESCs’ decidualization both in vivo and in vitro. With this purpose, for in vivo experiments, mice endometria were exposed to a high-fat/high-sugar diet (HF/HS) and were compared to those fed with standard chow, while for in vitro studies, immortalized human ESCs were obtained from the human uteri of normal weight and obese normo-ovulatory women and cultured in the presence or absence of palmitic acid (PA). It has been noted that the protein expression of phosphorylated acetyl-CoA carboxylase (*Acc*) and phosphorylated serine/threonine kinase *Ulk1*, two key regulators of autophagy in cells in their active form, were significantly lower in HF/HS-exposed mice suggesting that autophagy was upregulated more in decidualizing cells of the HF/HS-exposed mice compared to the control group. Furthermore, human ESCs displayed a markedly decreased mRNA expression of decidual prolactin (*PRL*) and insulin-like growth factor binding protein 1 (*IGFBP1*), two well-established decidualization markers in obese women compared to normal-weight women. Consistent with these data, it was also observed that the levels of *LC3B-II*, one of the best-characterized markers of autophagy, tended to increase with the decidualization in human ESCs than in controls. It is possible to deduce that autophagy, an essential factor in implantation, increases during the decidualization of human endometrial cells and is impaired by the exposure to excess fatty acid, contributing to obesity-related subfertility. Overall, these data may indicate that diet-induced obesity impairs ESCs’ decidualization both in vivo in an obese mouse model and in vitro human ESCs cultured model, potentially having both negative short-term effects on implantation and long-term fetal effects [31].

Obesity is additionally associated with an increase in adipose tissue due to the excessive caloric intake and significantly changes its structure and cellular composition. For this reason, it has been widely recognized that obesity-associated remodeling of the adipose tissue can equally cause a systemic proinflammatory state that is mediated by an imbalanced production of adipocytokines which directly and indirectly impair the reproductive system [32]. The adipocytokines are soluble mediators of adiponectin (ADIPOQ) derived from adipocytes and are mainly represented by leptin (LEP). Physiologically, these cytokines influence angiogenesis, controlling cell proliferation and differentiation, as well as inflammatory processes and immune tolerance by applying central or peripheral effects through their specific receptors, like the leptin receptor (LEPR) and adiponectin receptors 1 and 2 (ADIPOR1/R2) [33]. Different studies suggest that adiponectin, leptin and their corresponding receptors may be regulated at critical times in the peri- and post implantation periods at an endometrial level, resulting as crucial for endometrial gene expression and receptivity [34,35,36].

In addition, adipocytes along with myocytes are responsible for the production of the macrophage migration inhibitory factor (MIF). A study by Nahar A. and Kadokawa H. suggested that, in the oviducts of obese cows, the expression of the MIF is lower than for normal and lean ones. This molecule is important for regulating glucose absorption and glycolysis. MIF, influenced by the maternal nutritional condition, promotes specific functions for the ovum, sperm and embryos in the early stages of development in the oviduct, ovary and endometrium.

Insulin resistance, due to obesity, may explain the low expression of MIF in obese cows. In addition, an excess of energy sources in the maternal body can reduce the expression of MIF to protect the embryo. These results highlight the importance of maintaining an adequate nutritional status to ensure the correct expression of MIF and the consequent support of the reproductive processes in cattle [37]. 

Therefore, a study was conducted on the endometrial biopsies of women with unexplained recurrent implantation failure (IF) during the WOI compared to human endometria of fertile women, in order to assess the expression of adiponectin, leptin and their corresponding receptors between the two groups. It was evidenced that endometrial *LEP* expression was considerably lower, while the LEPR expression levels were higher in the endometria of the IF group in comparison to that of the respective fertile controls. By contrast, *ADIPOQ* was equally expressed in both groups, whereas the expression of the receptors, *ADIPOR1* and *ADIPOR2*, was reduced in the endometria of women with IF versus the ones of the fertile group. 

Furthermore, the higher serum leptin levels found in obese women are also reflected in the higher leptin levels in the follicular fluid. In vitro, leptin has been shown to influence the steroidogenic pathways in granulosa cells and to decrease dose dependently the oestradiol and progesterone production. The negative impact of obesity on the oocyte physiology may have downstream effects on the endometrial receptivity and embryo implantation [38].

Overall, these results may indicate that adiponectin and leptin signaling could act in an autocrine or paracrine manner to exert important roles in uterine receptivity and embryo implantation. Furthermore, even if implantation failure is a complex and multifactorial event, the down-secretion of leptin by the endometrium could be a biochemical cause behind female infertility, suggesting that restoration of leptin levels may be a new approach for the treatment of IF in women [34].

**Figure 1:**
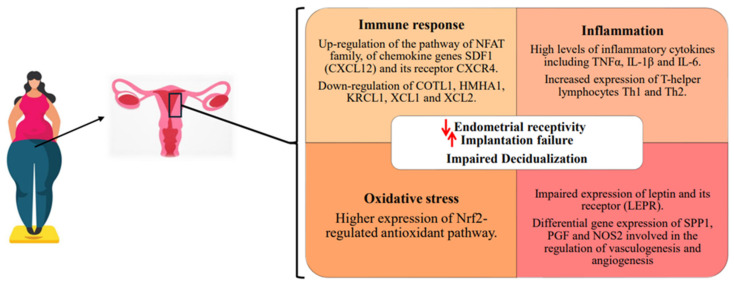
Primary pathways affected in mammals by obesity and its consequences on the endometrium. ↑ stands for increase, ↓ for decrease.

Wang et al. [39] aimed to evaluate the regulatory role of leptin in relation to four key genes for embryonic implantation, matrix metallopeptidase 9 (*MMP9*), heparin binding EGF-like growth factor (*HB-EGF*), β3-integrin (*ITGB3*) and *IL1B*, by exposing porcine endometrium epithelium cells (*PEECs*) to four different leptin concentrations (0.01, 0.10, 1.00, 10.00 nM) compared to non-exposed controls (0.00 nM). Following the culture in basal medium containing leptin for 24 h, it was observed that only 0.01 nM of leptin significantly improved *ITGB3* mRNA expression. In addition, *MMP9* and *HB-EGF* transcription were upregulated by 0.10–10.00 nM leptin, and the *IL1B* mRNA expression was increased by 10.00 nM leptin. Overall, *MMP9*, *HB-EGF* and *IL1B*, alongside their corresponding protein levels, exhibited a fluctuating response analogous to leptin elevation. By modulating the expression of *MMP9*, it could be sustained that leptin may be responsible for compromising porcine implantation. Upregulation of *HB-EGF*, on the other hand, could lead to an increase in vascular permeability that accelerates the degradation of stromal cells and potentially could be beneficial for the embryo invasion into the endometrium and for the exchange of maternal and fetal nutrients and metabolic wastes. Moreover, since the upregulation of *ITGB3* is provoked by a low concentration of leptin, this may potentially indicate that obesity, characterized by elevated levels of this hormone, may negatively affect uterine receptivity. In conclusion, behind the regulation of the *IL1B*-mediated pathways, there could be a participation by leptin in maternal-placental crosstalk during implantation. The current results indicate that leptin has a dose-dependent effect on the regulation of factors related to embryo implantation, and too high or too low leptin levels can both have disadvantages for the successful establishment of embryo implantation. Consequently, it is established that the alterations relating to leptin levels, observed in obesity, negatively influence not only endometrial receptivity but also implantation and therefore lead to lower fertility (Figure 1). 

### 2.2. Transcriptomic Profile of Endometrial Gene Alterations during the Window of Implantation in Infertile Obese Patients by Using the ERA-TEST

Until recently, one of the most used methods to examine endometrial receptivity has certainly been the endometrial receptivity analysis (ERA). The ERA-TEST is a specific microarray that studies the expression associated with 238 human genes. The state of endometrial receptivity is diagnosed thanks to the ERA computational predictor which pre-processes and normalizes the gene expression values, analyzing the specific window of implantation (WOI) to identify the ideal moment for embryo transfer.

**Figure 2:**
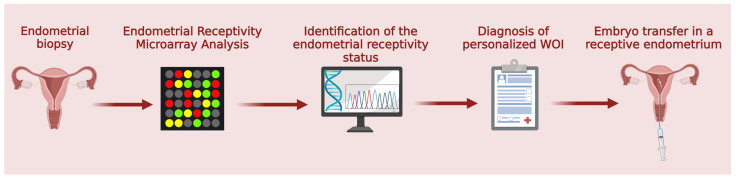
Scheme of the ERA-TEST workflow for the evaluation of endometrial receptivity and embryo transfer.

If the patient is receptive (R) during the expected window of implantation (eWOI), it is assumed that the transfer can be performed in another hormonal prepared cycle in the same period that the biopsy was performed. However, if ERA shows displacement of the window of implantation (dWOI), indicating that the endometrium is at the beginning or end of the receptive period, an adjustment will be required before embryo transfer [10] (Figure 2).

ERA was first used in patients with repeated implant failures. Afterwards, it was used in obese patients to analyze the endometrial functional genomics and to prove how this condition affects the endometrial transcriptomics related to genes that control metabolism. 

In the study by Bellver et al. [8], WOI shift rates were compared in 170 infertile women, with a normal uterus, divided into four groups with different body mass indexes (BMIs) and across obese (OB)/non-obese (NOB) categories. Thanks to the results for the endometrial receptivity obtained by ERA in a hormonally prepared cycle, it was found that the dWOI was dependent on the increase in BMI. The blood analysis of obese women showed high levels of insulinemia, LDL cholesterol, triglycerides, diastolic and systolic blood pressure, glycemia and thyroid stimulating hormone (TSH), while the levels of HDL cholesterol were significantly lower.

Thus, it could be suggested that the altered endometrial receptivity, linked to dWOI, is caused by the high BMI and the altered levels of the molecules involved in metabolism and endometrial receptivity functions. 

The ERA-TEST is used to evaluate the endometrial transcriptomic profile during the WOI thanks to its validity and accuracy in examining differential gene expression. For this reason, in order to determine the endometrial receptivity during the WOI, the ERA-TEST was also used on infertile obese patients and infertile normal-weight controls recruited and stratified into BMI categories according to the World Health Organization (WHO) guidelines [10]. The *COTL1*, *HMHA1*, *XCL2*, *XCL1* and *KLRC1* gene expression levels, for instance, were reported to be decreased in addition to a BMI increase. More specifically, *XCL1* and *XCL2* contribute to the positive regulation of chemokine activity and of extracellular signal-regulated kinase (ERKs) 1 and 2 pathways. The ERK pathways form a subfamily of mitogen-activated protein kinases (MAPKs) and regulate the fundamental cellular processes such as differentiation and proliferation. Therefore, in the WOI of obese patients, the ERK signal transduction is decreased, in line with *XCL1* and *XCL2* expression. This finding may potentially point to one of the mechanisms responsible for the increase in miscarriages and the reduction in implantation rates. On the other hand, three genes, *KRT7*, *MFAP5* and *S100A1*, were upregulated in obese receptive endometrial samples. These genes have functions of an extracellular structural nature and are related to the calcium binding matrix. It is therefore possible that changes in these expressions adversely affect the normal decidualization, a process defined by relevant changes in the cytoskeletal organization and extracellular matrix to allow placental trophoblastic invasion. As the BMI increased, a stronger incidence of non-receptive endometrium and higher gene expression were observed. The modulation in endometrial gene expression in obese women could be strongly linked to poor IVF outcomes and adverse pregnancy outcomes due to an increased infertility risk. 

In summary, it is evident that obesity, even in the absence of additional contributing conditions, can negatively affect female fertility due to an unfavorable intrauterine environment and reduced endometrial receptivity, leading to postponed WOI and worse ART outcomes.

## 3. Obesity and Endometriosis

Numerous physiological and pathological conditions can impact endometrial receptivity. For instance, hormonal imbalances have been linked to the failed implantation in polycystic ovary syndrome (PCOS) and endometrial receptivity. Endometrial pathologies such as endometriosis and polyps can raise the risk of pregnancy complications.

While endometriosis clearly affects fertility and endometrial receptivity as does obesity, the possible relationship between obesity and endometriosis is unclear. Obesity is associated with a proinflammatory environment which negatively impacts the endometrial receptivity and fertility. It is well established that inflammation plays a key role in the pathophysiology of the disease, impacting the immune cell function and elevating the production of proinflammatory cytokines in circulation, peritoneal fluid and endometrial tissues [40,41,42]. Endometriosis is a chronic, benign gynecological disease in which endometrial glands and stroma establish themselves in the peritoneal cavity and is associated with pelvic pain and infertility with detrimental effects on endometrial receptivity [18,19,20]. When surgically diagnosing endometriosis, a larger proportion of obese (defined as a body mass index [BMI] > 30 kg/m^2^) women were found to have an advanced, more severe disease, with a reduced number of these subjects having an early, minimal stage of the disease [43]. These observations might imply that obesity may increase the severity of the disease. Such a postulate could easily be tested in experimental models for endometriosis, but to date, few studies utilizing mice with experimentally induced endometriosis have been conducted. Heard and colleagues [44] conducted a study to examine this relationship in which they fed either a standard (SD; 17% total kcal from lard fat) or high-fat diet (HFD; 45% of total kcal from lard fat) to female mice from weaning to 56 post-natal days of age. Experimental endometriosis was then induced using endometrial tissue devoid of Krüppel-like factor 9 (*Klf9*), a key factor in progesterone receptor signaling. The consumption of HFD compared to SD did not augment the size of individual ectopic lesions, but it did increase the number of total lesions in mice fed HFD. Associated with the increased number of lesions in mice fed HFD was an increase in peritoneal fluid tumor necrosis factor-α (*Tnfa*), increased lesion cell proliferation, reduced apoptosis and increased macrophage infiltration into the lesion tissue. HFD also induced the lesion expression of a number of genes associated with immunity/inflammation including *Cxcl4* and *Il17a*. These observations suggested that (an HFD-induced-) inflammatory environment may predispose or augment the ability of endometriotic lesions to establish themselves and develop (Table 1). Accordingly, these observations would suggest that adiposity and an associated inflammatory environment may play a causative role in endometriosis rather than be a result of disease presence. In addition, increased adiposity might directly or indirectly affect the endometrial function. Obese women exhibit differential gene expression in the endometrium in association with endometrial cancer [12] as well as during the window of endometrial receptivity [10,45]. However, despite obesity having deleterious effects on the endometrial gene expression in these gynecological pathologies, a recent report by Holdsworth-Carson [46] failed to find any association between BMI and the altered endometrial gene expression in women with or without endometriosis. It should be noted in this study that the control population did not represent healthy controls and was composed of subjects suffering from pain symptoms sufficient to justify exploratory laparoscopy. Thus, further studies incorporating pain-free, healthy controls may be warranted to assess the relationship more thoroughly between obesity, endometriosis, and endometrial receptivity/fertility.

While these studies provide little support for a causative role/positive relationship of obesity/BMI in endometriosis, there is a substantial body of evidence that suggests that there is an inverse relationship between obesity/BMI and endometriosis. Ferrero and colleagues first reported that women with endometriosis have a lower BMI compared to women without the disease [47], which raised the question on the pathophysiology behind the decreased BMI and presence of the disease. Since then, several reports [48,49,50] have supported this inverse relationship between BMI/body size and the presence of endometriosis. One potential interpretation/explanation for these observations is that women with endometriosis who exhibit pelvic pain and a reduced quality of life may have less caloric intake and hence lower BMIs compared to women without endometriosis and associated symptomology [51].

This notion is supported by several studies which used mouse models of experimental endometriosis. Goetz and associates [52] induced experimental endometriosis in one group of mice, while a second group served as sham controls. All the mice were fed the same diet ad libitum and their body weights were assessed weekly while dual-energy X-ray absorptiometry (DEXA) was performed at 7 weeks post disease induction to assess the adiposity. While the mice in both groups increased in body weight over the 7-week timeframe, the control sham mice gained significantly more weight compared to the mice with experimental endometriosis. The DEXA assessment revealed that the endometriosis mice had significantly less total body fat as well as a lower percentage of body fat. To further explore the potential metabolic contribution to this lower weight gain and body fat composition, the hepatic gene expression was evaluated via microarray analysis. The induction of experimental endometriosis altered the hepatic expression of 26 genes (15 increased; 11 decreased compared to the sham controls), with six of these genes proposed to play a role in metabolism (elevated *Mrc1*, *Cyp2r1*, *Fabp4* and *Rock2*; decreased *Igfbp2* and *Mmd2*) (Table 1). 

**Table 1:** Overview of the experimental models, relative gene expression and potential biological outcomes included in the cited papers of paragraphs 2 and 3. In red, arrows that indicate up-regulation, while in green, arrows that indicate down-regulation.

Study Species	Genes/Pathways	Expression Modulation	Potential Biological Outcome	Reference
Human	*SDF1*, *TH1*, *TH2*	↑Upregulated	Compromised embryo implantation and embryo establishment	[22]
	*NRF2* pathway	↑Upregulated		
	*NFAT* pathway	↑Upregulated		
Human	*PRL*	↓Downregulated	Increased autophagy, impaired decidualization with potential negative effects on the implantation and fetus	[31]
	*IGFBP1*	↓Downregulated		
	*LC3B-II*	↑Upregulated		
Human	*LEP*	↓Downregulated	Alterations in uterine receptivity and embryo implantation	[34]
	*LEPR*	↑Upregulated		
	*ADIPOQ*	Not modulated		
	*ADIPOR1*	↓Downregulated		
	*ADIPOR2*	↓Downregulated		
Human	*COTL1*	↓Downregulated	Reduction in implantation rates, increased risk for miscarriages, impaired decidualization and potentially poor IVF outcomes	[10]
	*HMHA1*	↓Downregulated		
	*XCL2*	↓Downregulated		
	*XCL1*	↓Downregulated		
	*KLRC1*	↓Downregulated		
	*KRT7*	↑Upregulated		
	*MFAP5*	↑Upregulated		
	*S100A1*	↑Up-regulated		
Human	*AGE-RAGE*	↑Upregulated	Altered endometrial epithelial cell function, detrimentally impacted decidualization, promoted nuclear accumulation of NFκB and thus decrease in fertility	[15]
Ovine	*SPP1*	↑Upregulated	Alterations in implantation and placentation, compromised gestation, abnormal growth	[23]
	*PGF*	↑Upregulated		
	*NOS2*	↑Upregulated		
Murine	*Ccl2*	↑Upregulated	Uterine lipid deposition and extensive proinflammatory gene expression changes during the WOI	[28]
	*Ccl5*	↑Upregulated		
	*Ccl7*	↑Upregulated		
	*Cxcl10*	↑Upregulated		
	*Tlr2*	↑Upregulated		
	*Cd14*	↑Upregulated		
	*Ccr1*	↑Upregulated		
	*Lpl*	↑Upregulated		
	*Fabp4*	↑Upregulated		
	*Cd36*	↑Upregulated		
	*Rbp4*	↑Upregulated		
Murine	*Acc* (protein)	↓Downregulated	Increased autophagy, impaired decidualization with potential negative effects on the implantation and fetus	[31]
	*Ulk1* (protein)	↓Downregulated		
Murine	*Tnfa*	↑Upregulated	An inflammatory environment may predispose or augment endometriotic lesions, playing a causative role in endometriosis when associated with adiposity	[44]
	*Cxcl4*	↑Upregulated		
	*Il17a*	↑Upregulated		
Murine	*Mrc1*	↑Upregulated	Alteration in metabolism due to induced endometriosis	[52]
	*Cyp2r1*	↑Upregulated		
	*Fabp4*	↑Upregulated		
	*Rock2*	↑Upregulated		
	*Igfbp2*	↓Downregulated		
	*Mmd2*	↓Downregulated		
Equine	*TNFA*	↑Upregulated	Increased adiposity due to increased inflammation with a potentially compromised prenatal programming	[29]
	*IL1B*	↑Upregulated		
	*ATF6*	↑Upregulated		
Equine	*BAX* *CASP9* *P53* *P21* *OPN* *PI3K* *AKT* *BCL-2* *FOXP3* *SIRT1* *MFN1*	↑Up-regulated ↑Up-regulated ↑Up-regulated ↑Up-regulated ↑Up-regulated ↑Up-regulated ↑Up-regulated ↓Down-regulated ↓Down-regulated ↓Down-regulated ↓Down-regulated	Increased early apoptosis, loss of mitochondrial dynamics and the senescence-associated phenotype. The endometrium may be less prone to establishing an optimal environment for the embryo	[27]
Porcine	*MMP9*	↑Upregulated	Dose-dependent effect of leptin on the regulation of markers related to embryo implantation, with a consequent lower fertility potential	[39]
	*HB-EGF*	↑Upregulated		
	*ITGB3*	↑Upregulated		
	*IL1B*	↑Upregulated		
Bovine	*MIF*	↓Downregulated	These results highlight the importance of maintaining an adequate nutritional status to ensure support for the reproductive processes	[37]

In a follow-up study by this same research team [53], experimental endometriosis was induced in mice, but in this experiment, endometriosis and sham controls were fed with a high-fat diet (HFD, defined as 20% protein, 59.9% fat, 20.1% carbohydrates) for 30 days with body weights/weight gain and adiposity determined in both groups. The mice with experimental endometriosis had significantly lower body weights and decreased fat/body weight and fat/lean ratios compared to the sham controls fed a HFD. Associated with these outcomes was an increase in hypocretin/orexin neurons in the lateral hypothalamus. No significant difference in food intake was noticed between the sham and endometriosis mice despite the lower body weights and fat ratios in the mice with experimental endometriosis. Taken together, these findings support the notion that the induction of experimental endometriosis is associated with a lower body weight and adiposity and this affect appears to be manifested at both the level of the liver and lateral hypothalamus. Thus, as related to humans, the lower BMI associated with endometriosis may be due to an alteration in metabolism and/or hypothalamic signaling. 

Further support for the notion that a lower BMI may be a result, rather than a cause, comes from a caloric restriction study conducted by Yin and associates. In that study, the caloric restriction (CR) of the recipient mice (defined as 30% less calories consumed compared to ad libitum (AD) fed mice) impaired the development and growth of ectopic lesions by approximately 88% compared to lesions in the AD-fed recipients [54]. The caloric restriction in the recipient mice after the establishment of experimental endometriosis also reduced the lesion growth by 93% compared to the lesions in the AD-fed recipients. In both paradigms, the CR was associated with a reduced lesion cell proliferation, angiogenesis, steroidogenesis and fibrosis, but augmented autophagy. 

Although not assessed in this study, one may anticipate a dampened inflammatory milieu associated with the lower BMI which may have contributed to the poor growth and survival of the ectopic lesion tissue.

In summary, there still remains uncertainty concerning the cause-and-effect relationship between obesity/BMI and endometriosis as well as the potential impact of obesity and endometriosis on endometrial receptivity. This may stem from at least, in part, the complex relationship between the BMI and not only endometriosis, but the reproductive health as well [55]. The BMI is influenced by factors which can be controlled by individuals such as diet, physical activity and the environment as well as others which cannot be including developmental and genetic factors and aging. To dissect out the contribution of pain and pain-associated responses, future studies incorporating pain-free controls for the comparison to women with endometriosis (and pain) would be an ideal starting point to allow for a more thorough investigation of the potential impact on and relationship of obesity and endometriosis with endometrial function and fertility.

## 4. Conclusions

The prevalence of obesity is exponentially increasing among reproductive-aged women and can severely compromise their reproductive physiology by modulating the gene expression in a variety of cells and tissues, inevitably affecting the biological processes such as ovulation, endometrial development and receptivity and embryo implantation. 

Numerous studies involving obese women undergoing ART treatments have also shown that a rise in the BMI is correlated with decreased rates of implantation, pregnancy and live births [56,57]. However, the mechanisms responsible for these detrimental effects are not well understood.

The window of implantation is a critical timeframe, tightly interlinked with endometrial receptivity, that, in most cases, spans from three to six days during the secretory phase. In some inflammatory or anatomic conditions, though, this window can be narrowed, preventing normal implantation, which can result in the loss of pregnancy or infertility. From a molecular point of view, the negative effect of excess female weight during the WOI on the endometrial receptivity has been well established [8]. Obese women have been reported to present a different endometrial gene expression during the WOI compared to normal-weight women, which can become more pronounced when infertility is associated. Modulated genes can be involved in the biological functions such as transcriptional regulation and development, metabolic processes or even endometrial physiology dysfunction [58].

Moreover, the expression levels of the pathways related to the immune response, inflammation and reactive oxygen species production have been found to be significantly higher in the endometrial samples of women unable to physiologically conceive due to obesity compared to the ones of their non-obese counterparts, possibly contributing to the manifestation of further pathological states (Figure 1) [22]. 

Endometriosis appears to be one of the most common benign gynecological proliferations with distinct features of chronic inflammation in women [59]. Obesity equally seems to be associated with more advanced stages of this disease [60], even though the cause–effect relationship between them is, to this day, not yet entirely elucidated. Thus, the combined potential effect of obesity and endometriosis on endometrial function and fertility is still uncertain.

Due to the growing prevalence of obesity and its effects on female fertility and systemic health, urgent action is required globally both at an individual and a political level. One approach to limit its influence would be to reduce the presence of obesity itself. The use of health and social interventions, particularly those related to physical exercise and the quality of nutrition, could alleviate the biological disturbances during the peri-conception period, improving the reproductive outcomes. 

In conclusion, further investigations are required to better elucidate the mechanisms that govern embryo implantation at an endometrial level, which could be potentially useful for the generation of new strategies to overcome implantation failure and improve the pregnancy rates in obese women.
